# Pre-treatment predictors of attrition in a randomised controlled trial of psychological therapy for severe and enduring anorexia nervosa

**DOI:** 10.1186/1471-244X-14-69

**Published:** 2014-03-07

**Authors:** Ghada B Abd Elbaky, Phillipa J Hay, Daniel le Grange, Hubert Lacey, Ross D Crosby, Stephen Touyz

**Affiliations:** 1 NSW Institute of Psychiatry Fellow, University of Western Sydney, School of Medicine, Sydney, Australia; 2 School of Medicine, Centre for Health Research, University of Western Sydney, Sydney, Australia; 3 School of Medicine, James Cook University, Townsville, Australia; 4 Department of Psychiatry and Behavioural Neuroscience, The University of Chicago, Chicago, IL, USA; 5 Eating Disorders Research Team, St George’s University of London, London, UK; 6 Neuropsychiatric Research Institute, Fargo, ND, USA; 7 Department of Clinical Neuroscience, University of North Dakota, School of Medicine and Health Sciences, Grand Forks, ND, USA; 8 School of Psychology, University of Sydney, Sydney, Australia

**Keywords:** Anorexia nervosa, Attrition, Premature termination of treatment, Eating disorders, Dropout, Treatment

## Abstract

**Background:**

Attrition is common in the treatment of anorexia nervosa and its causes are complex and incompletely understood. In particular, its relationship with adaptive function and motivational stage of change has been little studied. This study aimed to (1) investigate and (2) compare the strength of associations between adaptive function, stage of change and other previously found factors such as illness sub-type and treatment attrition in women with severe and enduring anorexia nervosa (SE-AN).

**Methods:**

Participants were 63 adult women with SE-AN of at least 7 years duration who were enrolled in a multi-site randomized controlled trial conducted from July 2007 through June 2011. Treatment comprised 30 outpatient visits over 8 months of either Cognitive Behaviour Therapy for Anorexia Nervosa (CBT-AN) or Specialist Supportive Clinical Management (SSCM) both of which were modified for severe and enduring illness. Assessments were done at baseline, end of treatment, and 6 and 12 month post treatment follow-up. Demographic variables, duration of illness, specific and generic health related quality of life (QoL), eating disorder (ED) and mood disorder symptoms, social adjustment, body mass index (BMI), and motivation for change were assessed with interview and self-report questionnaires. Treatment attrition was defined as leaving therapy after either premature termination according to trial protocol or self-instigated discharge. Binary logistic regression was used to investigate relative strength of associations.

**Results:**

Those who did not complete treatment were significantly more likely to have the purging sub-type of anorexia nervosa and poorer ED related QoL. There were no significant differences between attrition and which therapy was received, educational level, and global ED psychopathology, stage of change, BMI, social adjustment, duration of illness or level of depression. The strongest predictors on multivariable analysis were ED QoL and AN-purging subtype.

**Conclusion:**

This study supported previous findings of associations between attrition and purging subtype. Furthermore, we found associations between a potentially important cycle of attrition, and poorer EDQoL, which has not been previously reported. Contrary to expectations we did not find an association with BMI, severity of ED symptoms, low level of motivation to change ED features, or level of education.

## Background

Attrition rates for anorexia nervosa (AN) are typically high compared to other mental health problems and other eating disorders, and are between 20.2% to 51% in inpatient samples and 29% to 73% in outpatient samples [1]. Furthermore, those who drop out of treatment for AN have a poor prognosis and an increased likelihood of rehospitalisation [1]. The causes of treatment non-completion are complex and incompletely understood. A critical review by Wallier et al. [2] of inpatient treatment drop-out in AN found weight on admission, anorexia nervosa-purging subtype, and the absence of depression were significantly related to attrition. However, comparisons across the seven studies selected in this review were difficult because of methodological variations in definition of attrition, sample composition, and the factors considered potential predictors. A larger and more comprehensive review [1] of 26 studies found no evidence that baseline eating disorder symptom severity, psychiatric co-morbidity or treatment issues affected dropout and concluded that factors associated with dropout were inconsistent due to methodological flaws and small sample sizes. The most consistent association was with the purging subtype of AN. There was also good evidence that two psychological traits (high maturity fear and impulsivity) and two personality dimensions (low self-directedness and low cooperativeness) were related to dropout.

We have since conducted a systematic review [3] which identified 13 further studies [4-16]. We also concluded that AN-purging subtype, personality traits, eating disorder features such as desired low body mass index (BMI), drive for thinness, dietary restriction and higher weight concerns and psychiatric co-morbidity, were associated with attrition. However, in two of eight studies that specifically examined eating disorder symptoms there were no associations between such features and attrition and little was known regarding other putative predictors of attrition such as motivational stage of change. This is despite the well-known ambivalence about treatment and low motivation for recovery in AN [17] due to at least in part the ego–syntonic quality of the illness i.e., the sense of accomplishment and moral virtue people derive from their pursuit of thinness. It has been argued for example that AN serves a functional purpose through providing identity and a sense of self-worth, despite its serious health risks [18]. Thus people often undertake treatment reluctantly and only in response to pleas from family or friends. This is in spite of poor psychosocial adjustment and health related quality of life, especially in severe and enduring anorexia nervosa (SE-AN).

In this study we aimed to first investigate associations between adaptive function (as reflected in health related quality of life and social adjustment), stage of change and other previously found factors such as illness sub-type and treatment attrition in women with SE-AN. We hypothesised that, prior to treatment higher levels of eating disorder symptoms, lower BMI, longer duration of illness, presence of purging behaviours, higher levels of depression, poorer social adjustment, lower level of education and poorer motivation to change would predict attrition. Our second aim was to explore the strength of association between attrition and health related quality of life, stage of change and other more established predictors of attrition.

## Methods

This study is a secondary analysis of data from a randomized controlled trial conducted at two intervention sites (i. University of Sydney and ii. St George’s Hospital at the University of London) and a data and coordinating centre (the University of Chicago) [19]. Sixty-three female participants were randomized to Cognitive Behaviour Therapy for Anorexia Nervosa (CBT-AN) or Specialist Supportive Clinical Management (SSCM). All participants met DSM-IV [20] criteria for AN, excluding criterion D (amenorrhea), for more than 7 years (thus also meeting DSM-5 criteria [21]). Participants received 30 50-minute individual treatment sessions provided over a period of eight months in an outpatient setting.

### Participants

**Figure 1:**
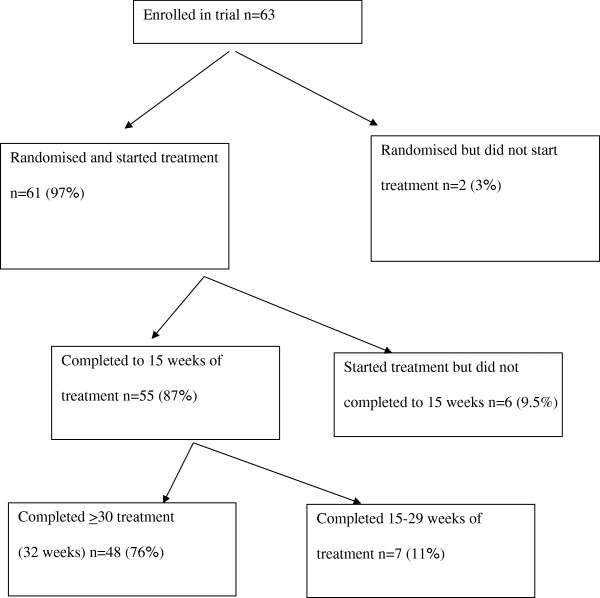
Participant flow through treatment #.

Participants were recruited from July 2007 to November 2010 by advertising to clinicians, clinics treating eating disorders (EDs), and on generic websites. After telephone screening (n = 159) to determine eligibility, 73 (46%) were invited for in-person assessment (see Figure 1). Respective site study coordinators described the protocol in detail to participants before written informed consent was obtained and the assessments conducted. Participants were eligible if they were female (males were excluded as we estimated that the number of such cases would be negligible), aged 18 years or more, met DSM-IV [20] criteria for AN, excluding criterion D (amenorrhea), and had an illness duration of at least 7 years. Participants were excluded from the study if they presented with a current manic episode or psychosis, current alcohol or substance abuse or dependence, significant current medical or neurological illness (including seizure disorder), with the exception of nutrition-related alterations that impact on weight, were engaged in psychotherapy and not willing to suspend treatment for the duration of their participation in the study, had plans to move beyond commuting distance from the study site in the following 12 months, or did not live within commuting distance to the study site. Eighty-six per cent (n = 63) of eligible participants agreed to randomization. The majority of those ineligible did not meet DSM weight loss [20,21] or trial illness duration criteria.

### Treatments

Participants were randomly assigned to either CBT-AN or SSCM by a biostatistician in the Data and Coordinating Centre at the University of Chicago, independent from either intervention sites. Randomization was conducted using Ephron’s biased coin approach, stratified within sites by subtype of illness (restrictive and binge-purge) and psychiatric medication status. To minimize therapist effect, three clinical psychologists with prior experience treating eating disorders in adults acted as therapists for both the CBT and SSCM conditions. All therapists attended two 2-day in-person workshops for training in the manualised treatments. All therapists treated pilot cases and were supervised by senior clinicians throughout the duration of the trial. Treatment occurred in outpatient settings at the University of London, and St George’s Hospital, University of London. Both treatments involved 30 individual treatment sessions provided over eight months.

SSCM includes education, care and support, while fostering a therapeutic relationship that promotes adherence to treatment. Supportive psychotherapy aims to assist the patient through use of praise, reassurance and advice. The rationale for this emphasis in treatment is that improvement in domains outside the core pathology can significantly affect patient well-being and disease burden, and research suggests that treatments that target psychosocial functioning are especially appropriate when there has been repeated relapse or a long duration of illness. Ultimately, SSCM aims to help individuals to improve their quality of life, which will further motivate and enable them to make progress on their core ED pathology.

CBT-AN and SSCM were both modified to prioritize quality of life and harm minimization associated with SE-AN, but in both weight gain was an important secondary aim. The primary outcome measures were selected to assess the extent to which individuals were better able to find satisfaction in their lives and engage meaningfully with significant others as a result of treatment. The treatments were distinct in that CBT-AN made use of specific cognitive and behavioural strategies whereas SSCM made use of a more collaborative and supportive therapeutic style.

### Assessment

Participants were assessed at pre-treatment and end of treatment by independent assessors blind to treatment assignment. Socio-demographic data were obtained by interview. Eating disorder features were assessed with the Eating Disorder Examination [22]. The EDE is a standardized investigator-based interview that measures the severity of the characteristic psychopathology of eating disorders. Studies consistently support its use, sensitivity, reliability and validity, making it the gold standard for assessing eating disorders. It includes investigator measurement of weight and height to calculate BMI (kg/m^2^).

Motivation to change was assessed with the Anorexia Nervosa Stages of Change Questionnaire (ANSOCQ) [23]. The ANSOCQ is a 20-item multiple choice questionnaire that assesses a patient’s readiness to recover from AN. It has demonstrated good validity and has high levels of inter-rater and test–retest reliability.

Eating disorder related quality of life was assessed with the Eating Disorder Quality of Life questionnaire (EDQoL) [24]. The EDQoL is a standardized and validated 25-item instrument assessing quality of life in eating disorder populations across four subscales: psychological, physical and cognitive, financial, and work or school. This is the first instrument designed to assess quality of life in eating disorder patients. Because of an inherent bias in existing instruments towards assessing occupational attainment, it is often a challenge to accurately evaluate quality of life in this population. This instrument is uniquely designed to assess relevant components of quality of life for individuals with anorexia nervosa and has demonstrated reliability and validity.

General health related quality of life was assessed with the Short Form-12 Health Status Questionnaire (SF-12) [25]. The well validated SF-12 measures dimensions of health and role limitations due to physical and mental health, for which Mental (MCS) and Physical Component Summary (PCS) scales can be derived.

Depression was assessed with the Beck Depression Inventory (BDI) [26]. The BDI is a 21–question scale with each answer rated 0–3, this scale is widely used across the entire spectrum of studies of psychopathology and psychotherapy.

Social adjustment was assessed with the Weissman Social Adjustment Scale (WSAS) [27] The WSAS assess social adjustment in multiple areas of functioning, including marital, family, work, economic and leisure. The scale has well established reliability and validity and has been used in a wide variety of populations.

### Attrition

Previous reviews e.g. [1,3] have not identified an agreed definition of treatment completion. Furthermore, the small sample size of the present study precluded separate analyses of treatment non-engagement (randomised but did not engage in treatment), early attrition (in this study defined as less than 15 weeks of treatment competed, the definition used in the main report of the trial [19] (p.6) and late attrition (in this study defined as completing 15–29 weeks of treatment). As shown on Figure 1, two participants were randomised but failed to engage in treatment, 55 participants completed at least 15 weeks of treatment (as reported previously [19]), and 48 participants completed 30 or more weeks of treatment.

### Statistical analyses

Data were analysed using SPSS v 10. Data were inspected for normality. Univariate analyses were conducted on categorical data using Chi-square test (with Yates correction for small cells), and on continuous data using *t*-test for parametric data and Mann–Whitney *U* test for non-parametric data. The alpha level was corrected for multiple testing to ≤ 0.01. Binary logistic regression analysis was used to model the predictors of treatment dropout that were identified in the univariate analyses, namely duration of anorexia nervosa, AN-subtype, global EDE score, EDQoL total and WSAS scores. (Logistic regression was dictated by the dichotomous outcome. More sophisticated multivariable analyses, such as structural equation modelling, were precluded by the insufficient sample size). The model was run with forward entry where the best variable is entered, then the next, etc., until no more variables significantly contribute to the prediction. The model was then run with backward entry whereby all variables are forced in, and one-by-one non-significant variables are removed. As the final results did not differ, only the forward entry model is reported in this paper.

## Results

A total of 63 participants were randomly assigned to CBT-AN (n = 31), or SSCM (n = 32). All study participants were female and ranged in age from 20 to 61 years. As reported in Table 1 participants had severe and enduring illness (as reflected in the mean global EDE score of 2.78 and mean duration of illness of 14 years) and very poor health related quality of life (as reflected in mean SF-12 mental health component score of 31.88). Applying DSM-5 [21] definitions of severity based on BMI, the mean BMI of this sample was in the moderately severe range i.e. between 16 and 16.99.

**Table 1:** Demographic and clinical features of those who completed treatment and those who did not complete treatment

**Feature**	**Completers**	**Non completers**	**Total**	**Statistical result**
	**n = 48**	**n = 15**	**n = 63**	
	n (%)			*χ*^2^, df, p
Anorexia Nervosa Purging type	8 (16.6%)	8 (53.3%)	16 (25.4%)	8.11, 1, 0.008*
Graduate education	32 (67%)	8 (57%)	40 (64%)	0.43, 1, 0.36
Randomised to CBT therapy	22 (46%)	9 (60%)	31 (49%)	0.92, 1, 0.34
	Mean (SD)			t, df, p
EDE global score	2.78 (1.28)	3.70 (1.45)	3.00 (1.37)	2.34, 61, 0.01
EDE restraint score	2.86 (1.81)	3.90 (1.27)	3.11 (1.75)	2.05, 61, 0.023
EDE eating concern score	2.62 (1.49)	3.28 (1.54)	2.78 (1.52)	1.49, 61, 0.07
EDE shape concern score	3.08 (1.36)	4.04 (1.71)	3.31 (1.50)	2.23, 61, 0.015
EDE weight concern score	2.56 (1.62)	3.57 (1.96)	2.80 (1.75)	2.01, 61, 0.024
SF 12 physical health component score	48.55 (9.70)	50.29 (6.75)	48.99 (9.03)	0.87, 61, 0.19
SF 12 mental health component score	33.39 (12.55)	27.44 (10.20)	31.88 (12.20)	−1.49, 61, 0.07
BDI total Score	25.04 (14.10)	30.44 (12.59)	26.41 (13.84)	1.20, 61, 0.12
EDQOL psychological score	2.53 (0.75)	3.08 (0.67)	2.66 (0.77)	2.53, 61, 0.007*
EDQOL physiological cognitive score	2.09 (0.88)	2.31 (0.31)	2.14 (0.78)	0.88, 61, 0.19
EDQoL Work & school score	0.61 (0.68)	1.29 (0.65)	0.78 (0.73)	3,18, 52, 0.002*
EDQoL Financial score	0.48 (0.69)	1.14 (0.71)	0.63 (0.74)	3.12, 61, 0.002*
EDQoL Total score	1.67 (0.60)	2.21 (0.38)	1.81 (0.60)	3.06, 61, 0.002*
Work and Social Adjustment Scale Total score	16.98 (10.36)	22.69 (8.05)	18.43 (10.08)	1.94, 61, 0.029
ANSOQ Total score	2.59 (0.58)	2.48 (0.63)	2.56 (0.59)	0.89, 61, 0.18
ANSOCQ Weight gain score	2.19 (0.73)	1.98 (0.79)	2.14 (0.74)	1.11, 61, 0.14
ANSOCQ Eating, Weight and Shape score	2.82 (0.66)	2.77 (0.58)	2.81 (0.64)	.44, 61, 0.32
ANSOCQ Ego –Alien Aspects score	2.79 (0.69)	2.72 (0.77)	2.77 (0.71)	.591, 61, 0.27
	Median (IQ range)			Mann–Whitney UZ, p
Duration of illness/years	14 (9–19.8)	19 (14–26)	14 (9–23)	1.74, 0.041

Probability levels are one-tailed, *p < 0.01, CBT = Cognitive Behaviour Therapy, EDE = Eating Disorder Examination [22], SF = Short-Form [25], BDI = Beck depression inventory [26], EDQoL = Eating Disorder Quality of Life [24], ANSOCQ = Anorexia Nervosa Stage of Change Questionnaire [23].

Rates of treatment completion did not differ by site (23, 79%, completed treatment in Sydney and 25, 74%, in London) and this difference was not significant (*χ*^2^ = 0.768, df = 1, p = 0.41). Demographic and clinical features of the sample and comparisons between those who did and did not complete treatment are found in Table 1. There were no differences between the two therapy groups and treatment completion. Those who did not complete treatment were significantly more likely to be of AN-purging subtype and to have poorer EDQoL. There were no significant differences between attrition and which psychological therapy was received, educational level, motivational stage of change, BMI, global eating disorder psychopathology. general health related quality of life, social adjustment, duration of anorexia nervosa or level of depression.

The results of the logistic regression are found in Table 2. With forward entry the variables EDQoL total score (OR = 0.197 95% CI 0.047; 0.832, p = 0.027) and AN-subtype (OR = 0.259, 95% CI 0.067; 1.009, p = 0.051) were retained in the model. However, the OR CI for AN-subtype included 1.0 and just failed to reach significance.

**Table 2:** Results of multivariable (logistic regression forward) analyses with treatment completion as dependent variable

**Predictor (independent) variable**	**Nagelkerke R**^**2**^	**Odds ratio**	**95% C.I.**	**p (variable)**	**p (overall model)**
Step 1:	0.216	0.144	0.035; 0.603	0.008	0.002
ED Quality of Life Total score [24]					
Step 2:					
ED Quality of Life Total score [24]	0.292	0.197	0.047; 0.832	0.027	0.001
AN-purging subtype	-	0.259	0.067; 1.009	0.051	

Note: Variables not in the final equation were duration of illness (p = 0.13), global Eating Disorder Examination [22] (p = 0.58) and Weissman Social Adjustment Scale scores [27] (p = 0.33).

## Discussion

This study aimed to first to investigate associations between health related quality of life, stage of change and other previously found factors such as illness sub-type and treatment attrition in women with SE-AN, and the second aim was to explore the strength of association between adaptive function and stage of change and other more established predictors of attrition. In this study, as in Halmi et al. [28] there were few factors associated with treatment dropout; one of which was however not previously reported namely, poor eating disorder quality of life. The previously consistently identified factor of AN-purging subtype [1-3] was as well associated with treatment attrition in this study. There were no significant differences between attrition and therapy randomized to, educational level, motivational stage of change, BMI, global eating disorder psychopathology, general health related quality of life, social adjustment, duration of illness or level of depression. Furthermore, the strongest predictors on multivariable analysis were eating disorder quality of life and AN-purging subtype.

Our findings are consistent with many others that have reported AN-purging subtype to predict treatment attrition [1,2] in both inpatients and outpatient setting. We similarly found the EDE global and subscale scores to be associated with higher a level of attrition which supports the majority of previous studies. Thus, the more severely ill participants, in terms of behavioural, social and psychological parameters, the more likely they are to drop out of treatment, and this was unaffected by the specific psychological therapy they received. Clinicians should take particular effort to engage and retain these patients in care, as they have more severe illness and whilst in most need of care are more likely to terminate prematurely.

To our knowledge, this is the first study to find that overall eating disorder specific quality of life predicted attrition. In addition overall specific quality of life was the strongest predictor in the multivariable model in this study. As persistent anorexia nervosa most often over time is associated with an increase in social and interpersonal deficits eating disorder quality of life may be an even stronger predictor in studies where there is more variability in illness duration and adaptive function. Our findings highlight the importance of further investigation of the relationship between this and attrition, and interventions to prevent and/or break a cycle of functional decline and premature termination of treatment. The generic measure of quality of life and eating disorder specific quality of life in the physical and cognitive domains (as compared to financial, psychological, work and schooling) did not however predict attrition. This may be because the ego-syntonic nature of anorexia nervosa is associated with denial or minimisation of the physical and cognitive effects of illness.

Whilst depression was associated with higher level of attrition this was not statistically significant, and depression has not consistently been associated with attrition in other studies. For example, Masson et al. [29] reported that co-morbidity with depression in fact enhanced treatment completion in the inpatient setting. However, it is possible the study lacked statistical power to detect a difference. This may also apply to the findings for global eating disorder psychopathology, social adjustment and duration of illness where there appeared to be associations that however failed to reach significance. Caution should thus be applied to these negative findings. The lack of statistical association between preadmission BMI and premature treatment termination that we found may have been because this was an outpatient trial and BMI was not as low or as variable as BMI as in other studies. However, most other studies have also not found an association with BMI and attrition.

Our findings did not support those of Dalle Grave et al. [30] and Huas et al. [12] who found an association between level of education and attrition. This may have been because of the relatively high level of graduate education (67%) and low level of variability in education level in the present study participants.

Findings continue to be inconsistent with regard to the few studies investigating level of motivation and treatment attrition. In our study we did not confirm our hypothesis that stage of change would predict treatment attrition. This may have been because the motivation to participate in this study was for the stated primary aim of improved quality of life rather than weight gain (although weight gain was clearly an important secondary aim). However, the ANSOCQ questionnaire measures motivation related to reduction in eating disorder behaviours and increase in body weight, not change in quality of life. The relationship between stage of change and various measure of treatment outcome is also very complex. For example, whilst finding no relationship between stage of change and BMI outcome, Mander at al. [31] found being at the stage of contemplation predicted treatment alliance.

The strengths of this study are the comprehensive range of predictors and validated assessment instruments. The major limitations are the small sample and unequal groups (n = 15 non completers vs. 48 completers) which increased the likelihood of Type II statistical error, precluded separate analyses of participants with early and later attrition and examination of bidrectionality of relationships using more sophisticated analytic methods such as structural equation modelling. Unequal group sizes and small numbers in the attrition arm were the results of the low rates of dropout in the present study. This contrasts with other outpatient studies, where completion rates can be as low as 27% [1]. A more recent outpatient trial of Zipfel et al. [32] also reported a treatment completion rate of 74% but Schmidt et al. [33] reported that only 25% participants completed the majority (>61%) of sessions. The low rates in the present may have been because of the modification of treatment goals and tailoring of treatment to the participant’s stage of change. Finally, co-morbidity more broadly, i.e. personality characteristics, could not be examined in the present study. The findings as well may not generalise to participants with shorter illness duration.

## Conclusion

As expected, AN-purging subtype was associated with a higher treatment attrition rate. A new finding was the association with eating disorder specific overall quality of life. However, particularly given this study’s small sample size, further study is needed to investigate the likely bidirectional nature of declining adaptive function and increase risk of treatment dropout in this serious illness. More research is needed to test our supposition that people with long standing anorexia nervosa may be more motivated to engage in treatment where goals are modified from primacy of weight regain to improved quality of life. Potentially therapy should be also further modified to address the needs of those with greater deficits in adaptive function.

## Abbreviations

SE-AN: Severe enduring anorexia nervosa; CBT: Cognitive behavioural therapy; SSCM: Specialist supportive case management; EOT: End of treatment; BMI: Body mass index; ED: Eating disorder; DSM: Diagnostic and statistical manual of mental disorders; ANSOCQ: Anorexia nervosa stage of change questionnaire; EDQoL: Eating Disorder Quality of Life; SF-12: Short form health status questionnaire; MCS: Mental health component score; PCS: Physical health component score; BDI: Beck Depression Inventory; WASAS: Weissman Social Adjustment Scale; EDE: Eating disorder examination.

## Competing interest

The authors declare that they have no competing interests.

## Authors’ contributions

ST, HL, and DleG were investigators in the parent randomised controlled trial, provided intellectual input into this study design and reviewed the final manuscript. GA conducted the background literature search wrote the manuscript and conducted analyses supervised by PH. PH was an investigator in the parent randomised controlled trial, provided intellectual input into this study design, conducted analyses, and was involved in drafting and revising the manuscript. RG advised on study design, performed the multivariable statistical analyses and was involved in drafting and revising the manuscript. All authors read and approved the final manuscript.

## Pre-publication history

The pre-publication history for this paper can be accessed here:

http://www.biomedcentral.com/1471-244X/14/69/prepub
